# Implementation of Cardiac Computed Tomography for Aortic Valve Infective Endocarditis: Coronary Arteries Evaluation and Concordance with Transoesophageal Findings

**DOI:** 10.3390/diagnostics16142160

**Published:** 2026-07-10

**Authors:** Pietro Valsecchi, Katia Ellena, Alessandro D’Onorio De Meo, Adele Valentini, Roberta Simona Cattaneo, Calogero Volpe, Filippo Amoroso, Catherine Klersy, Giulia Magrini, Stefano Pelenghi, Raffaele Bruno, Elena Seminari

**Affiliations:** 1 Unità Operativa Malattie Infettive I, Fondazione IRCCS Policlinico San Matteo, Viale Golgi, 19, 27100 Pavia, PV, Italy; 2 Unità Operativa Radiologia Diagnostica per Immagini 1, Fondazione IRCCS Policlinico San Matteo, Viale Golgi, 19, 27100 Pavia, PV, Italya.donorio@smatteo.pv.it (A.D.D.M.);; 3 Unità Operativa Cardiologia, Fondazione IRCCS Policlinico San Matteo, Viale Golgi, 19, 27100 Pavia, PV, Italy; 4 Unità Operativa Cardiochirurgica I, Fondazione IRCCS Policlinico San Matteo, Viale Golgi, 19, 27100 Pavia, PV, Italy; 5 SSD Biostatistica e Clinical Trial Center, Fondazione IRCCS Policlinico San Matteo, Viale Golgi, 19, 27100 Pavia, PV, Italy; 6 Dipartimento di Scienze Clinico-Chirurgiche, Diagnostiche e Pediatriche, Università degli Studi di Pavia, Viale Golgi, 19, 27100 Pavia, PV, Italy

**Keywords:** cardiac CT, transoesophageal echocardiography, aortic valve, infective endocarditis, coronary artery

## Abstract

**Background:** Cardiac computed tomography (CCT) is recommended as a complementary exam and for coronary artery disease evaluation in patients with aortic valve IE (AVIE). We aimed to evaluate the performance of CCT in visualizing coronary arteries in patients with AVIE and its concordance with transoesophageal findings. **Methods:** We included patients admitted for AVIE who underwent CCT from 2016 to 2023. Coronary arteries were divided into 17 segments according to the American Heart Association and compared using generalized linear models extended to the binomial family. Results were presented as risk differences (RDs) and 95% confidence intervals (CIs). For those undergoing cardiac surgery, we evaluated the concordance among CCT, TOE, and surgical inspection using Cohen’s kappa (κ) test. **Results:** During the study period, 39 patients with AVIE performed CCT, of whom 28 underwent cardiac surgery. Visibility of coronary arteries was lower in median segments (RD −0.23; 95% CI −0.32 to −0.14) and in distal segments (RD −0.25; 95% CI −0.36 to −0.15) compared to proximal ones. Visibility of left anterior descending artery 2 was higher compared to right coronary artery 2 (RD 0.33; 95% CI 0.07 to 0.45) and obtuse marginal (RD 0.33; 95% CI 0.52 to 0.15). Concordance between CCT and TOE was globally low for vegetations (kappa 0.14), pseudoaneurysms (kappa 0.46), and paravalvular leakage (kappa 0.36). **Conclusions:** This study highlights the complementary role of CCT in diagnosing IE, given its low concordance with TOE for specific IE lesions. Furthermore, while CCT offers good visibility of the proximal coronary arteries in patients with AVIE, the visibility of the median and distal segments may be suboptimal.

## 1. Introduction

Infective endocarditis (IE) is a severe disease characterized by a high mortality rate despite diagnostic and therapeutic innovations [1]. While blood cultures and transoesophageal echocardiogram (TOE) represent the mainstay of IE diagnosis, cardiac computed tomography (CCT) has emerged as a complementary diagnostic instrument in IE and has been recently included as a major diagnostic criterion in the updated version of the Duke criteria by the International Society of Cardiovascular Infectious Disease (ISCVID) [2]. The 2023 European Society of Cardiology (ESC) guidelines recommend CCT for patients with possible IE to detect paravalvular complications and for those with an inconclusive TOE for whom IE is strongly suspected [3]. These lesions, such as abscesses and pseudoaneurysms, are associated with increased mortality in patients with IE [4].

According to a recent systematic review and meta-analysis, CCT had a sensitivity of 88% for detecting paravalvular complications compared with 70% for TOE [5]. Conversely, TOE had higher sensitivity than CCT for vegetation (96% versus 85%), leaflet perforation (79% versus 48%), and paravalvular leakage (100% versus 85%).

While previous studies focused on the different diagnostic performance of TOE and CCT, data on concordance between the two imaging modalities are lacking.

Furthermore, computed tomography angiogram (CTA) is strongly recommended for the evaluation of coronary artery disease (CAD) in patients with aortic valve IE (AV-IE) and surgical plan, since preoperative coronary angiography (CA) is usually contraindicated due to increased risk of iatrogenic embolization in the coronary arteries [3,6,7]. CTA has shown a good negative predictive value (>90%) for ruling out CAD in stable patients [8], but data on patients with AV-IE are limited [7]. In particular, one possible limitation is that these patients are frequently tachycardic due to sepsis and/or heart failure, thus evaluation of the coronary arteries may be limited due to motion [9].

A suboptimal cardiac CT may result in being inconclusive while exposing the patient to radiation and to the risk of contrast-induced allergic reactions and nephrotoxicity [10]. This is even more concerning considering that patients with IE frequently present with concomitant chronic or acute kidney failure, which is associated with poor clinical outcomes [11].

With this in mind, we aimed to evaluate the feasibility of CTA to describe coronary arteries and to diagnose CAD in a cohort of patients with AV-IE. Secondly, we aimed to assess the diagnostic concordance between cardiac CT and TOE for IE, using intraoperative findings as the standard reference.

## 2. Materials and Methods

Data from consecutive patients admitted to IRCCS Fondazione Policlinico S. Matteo from the 1 January 2016 and the 31 March 2024 with a definite IE diagnosis were retrieved from the local endocarditis registry (STEADY registry, Internal review board approval P-20200060). Patients were included if they had aortic valve IE (AV-IE) and had undergone a CCT during the IE-related hospitalization. Exclusion criteria were IE affecting valves other than the aortic valve and/or absence of CCT.

IE diagnosis was defined according to modified versions of the Duke criteria. Serum creatinine levels were obtained from the last exam performed before Cardiac CT, and the estimated glomerular filtration rate (eGFR) was calculated using the Chronic Kidney Disease Epidemiology Collaboration (CKD-EPI) equation [11]. Acute kidney injury (AKI) was defined according to the Kidney Disease Improving Global Guidelines (KDIGO) definitions. It was considered related to cardiac CT if it occurred within 72 h after the exam [12]. Allergic reactions were defined as nausea, vomiting, mild urticaria, pallor, severe vomiting, extensive urticaria, dyspnea, rigor, laryngeal edema, or circulatory collapse occurring after infusion of iodine contrast enhancement [11]. For patients with a known history of allergic reactions to contrast enhancement, preparation was performed in accordance with local standard procedures.

**Figure 1:**
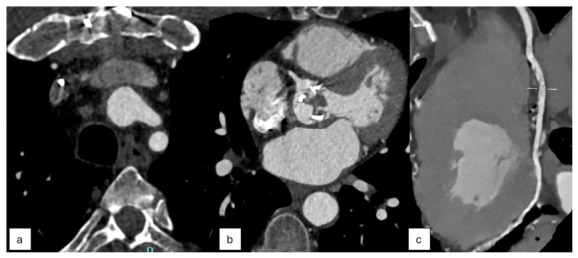
Examples of lesions detected by CCT: (**a**) paravalvular abscess surrounding the aortic root in a patient with prosthetic valve endocarditis, (**b**) a case of endocarditis involving a mechanical prosthetic aortic valve complicated by a pseudoaneurysm, (**c**) CTA showing an atherosclerotic plaque involving the right coronary artery 2.

For patients who underwent surgical intervention, the presence of cardiac lesions characteristic of IE (vegetation, leaflet perforation, abscess, leakage, and pseudoaneurysm) as detected by CCT, TOE, and surgery was defined according to the 2023 ESC Guidelines for infective endocarditis (Figure 1) [3].

### 2.1. Study Outcomes

The primary endpoint was the ability of CCT to visualize coronary arteries and detect critical lesions according to heart rate and the presence of atrial fibrillation or extrasystoles. Secondary endpoints were: (i) concordance between CCT and TOE for vegetation, leaflet perforation, abscess, leakage, and pseudoaneurysm, using surgical report as the reference standard; and (ii) prevalence of AKI and allergic reactions related to CCT.

### 2.2. Radiological Methods

Two radiologists independently reviewed all ECG-gated CT angiograms (CTAs) and related reports performed at Fondazione IRCCS Policlinico San Matteo during the study period in patients diagnosed with aortic valve infective endocarditis and with an indication for cardiac and coronary artery imaging. A third radiologist resolved any discrepancy.

CTAs were performed on MDCT systems Siemens Somatom Definition (Siemens Healthineers, Erlangen, Germany), Toshiba Acquilone One (Canon Medical Systems, Ōtawara, Japan) and Siemens Healthineers Somatom go.Top (Siemens Healthineers).

A 3-phase acquisition protocol was used. First, a non-contrast-enhanced acquisition was performed. Secondly, an ECG-synchronized CTA was acquired, with images reconstructed in diastole and systole. Third, a delayed phase acquisition covering the entire chest (and in specific cases, including the abdomen or brain as well) was acquired.

An institutional protocol instructed clinicians requesting ECG-synchronized CTA to lower the patient’s heart rate before the exam using bisoprolol, diltiazem, or ivabradine. During the exam, β-blockers and nitroglycerin were not routinely administered but were considered in selected cases to lower heart rate when relevant contraindications were absent.

For each CTA, heart rate, rhythm anomalies, possible premedication, and possible allergic reaction to the contrast medium were evaluated.

IE findings collected include valvular vegetations with their sizes, valve perforations, perivalvular complications such as abscesses and pseudoaneurysms with their respective numbers, fistulas, and mitral-aortic fibrosa involvement. The presence of paravalvular leakage was also investigated in patients with prosthetic valve infective endocarditis.

For the evaluation of the coronary arteries, the AHA classification [13] was used, and for each of the 16 segments, the visibility and possible presence of significant lesions were scored. The presence of stents and coronary artery bypass grafts has also been reported.

The coronary segments were then divided into proximal segments (proximal segment of the right coronary artery (RCA1) (1), common trunk of the left coronary artery (LM) (5), proximal segment of the anterior interventricular artery (LAD1) (6) and proximal segment of the circumflex artery (LCX1) (11), middle segments (intermediate segment of the right coronary artery (RCA2) (2), intermediate segment of the anterior interventricular artery (LAD2) (7) and obtuse marginal branch (OM) (12) and distal segments (distal segment of the right coronary artery (RCA3) (3), distal segment of the anterior interventricular artery (LAD3) (8) and distal segment of the circumflex artery (LCX2) (13) to investigate the quality of CTAs in patients with aortic valve IE.

Transesophageal echocardiography (TOE) was performed using an X7-2t Live 3D multi-plane probe (Philips Healthcare, Andover, MA, USA). The recorded echocardiographic images were retrospectively reviewed for diagnostic accuracy. The presence of infective endocarditis lesions was assessed in standard esophageal and transgastric views, with additional non-standard imaging planes used when necessary for improved visualization, particularly in patients with prosthetic valves. In all cases, a brief transthoracic echocardiogram was also performed to evaluate biventricular size and function.

### 2.3. Statistical Analysis

All statistical analyses were performed using Stata 19.5 (StataCorp, College Station, TX, USA). Qualitative variables were described as counts and percentages of each category. Quantitative variables have been summarized as median and interquartile range (IQR) or as mean and standard deviation (SD), depending on the distribution. A two-sided *p*-value < 0.05 was considered statistically significant. The Bonferroni correction was applied for post hoc comparisons.

The proportion of visible coronary arteries was computed across all segments, along with its exact binomial 95% confidence interval (95% CI), both overall and by level. Comparisons between coronary artery segments were made using generalized linear models with a binomial distribution, and results were reported as risk differences (RDs) with 95% confidence intervals (CIs). Huber-White robust standard errors were computed to account for intra-patient correlation of measures.

For those undergoing cardiac surgery, we evaluated the agreement among CCT, TOE, and surgical inspection using Cohen’s kappa.

An optimal cut-off for heart rate during CCT, maximizing sensitivity and specificity for non-visibility of the coronary arteries, was computed from the area under the ROC curve (AUC-ROC) of the sensitivity vs. 1-specificity plot.

## 3. Results

### 3.1. Demographic and Clinical Characteristics

During the study period, 381 patients were diagnosed with IE, of whom 216 had AV-IE. Thirty-nine patients with AV-IE underwent a CCT, and of these, 28 required cardiac surgery.

Six of the included patients (15.4%) were female, and the median age was 65 years (IQR 54–73). Complete demographic and clinical characteristics of the included patients are shown in Table 1.

A previous surgical procedure was recorded in 21 patients (53.9%), and 20 (52.6%) had a prosthetic valve IE. A stent was in place in 3 patients (7.69%), and 3 (7.69%) had previously undergone a bypass procedure.

*Staphylococcus* spp. was the most frequent bacterial genus recovered from blood cultures in 17 cases (41.03%), of which 7 were *Staphylococcus aureus*; *Enterococcus faecalis* was recovered in 12 (30.77%) cases, and *Streptococcus* *spp.* in 9 (23.08%) cases. Detailed information regarding causative pathogens and antimicrobial treatment is provided in Table S1.

Median times between TOE and CCT, and between CCT and cardiac surgery, were 6 days.

### 3.2. Coronary Arteries Evaluation

**Figure 2:**
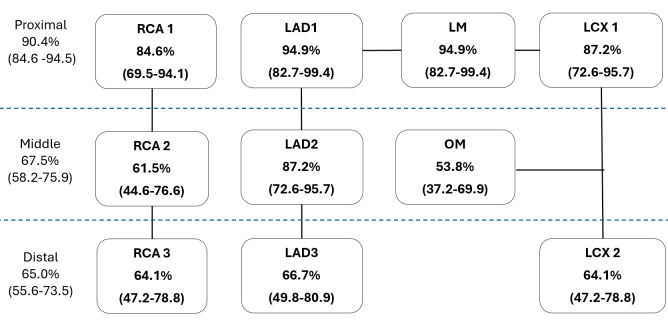
Coronary artery visibility by segment; results are expressed as proportions and 95% CIs. RCA = right coronary artery, LAD = left anterior descending, LM = left main, OM = obtuse marginal, LCX = left circumflex.

Evaluation of the coronary arteries was performed on 39 patients and 558 segments. Globally, 391/558 segments were visualized (prevalence 70.1%, 95% CI 66.1–73.8). The proportion of visible coronary arteries by coronary segment is shown in Figure 2.

Generalized linear models showed lower visibility of coronary arteries in median segments (RD −0.23; 95% CI −0.32 to −0.14) and distal segments (RD −0.25; 95% CI −0.36 to −0.15) compared with proximal segments.

Visualization was similar within proximal segments, in particular between right coronary artery 1 (RCA1) and left main (RD 0.10; 95% CI −0.02 to 0.22), between RCA1 and left anterior descending artery 1 (RD 0.10; 95% CI −0.02 to 0.22) and between RCA1 and left circumflex 1 (RD 0.03; 95% CI −0.13 to 0.18). Post hoc pairwise comparisons of marginal linear predictions with a Bonferroni correction yielded similar results.

Visibility of left anterior descending artery 2 (LAD2) was higher compared to right coronary artery 2 (RD 0.33; 95% CI 0.07 to 0.45) and obtuse marginal (RD 0.33; 95% CI 0.52 to 0.15). This difference was confirmed after performing post hoc pairwise comparisons of marginal linear predictions with a Bonferroni correction.

With regard to distal segments, visibility was similar between right coronary artery 3 (RCA3) and left anterior descending artery 3 (RD 0.03; 95% −0.13 to 0.18) and between RCA3 and left circumflex 3 (RD 0.0; 95% CI −0.18 to 0.18). Post hoc pairwise comparisons of marginal linear predictions with a Bonferroni correction yielded similar results.

When evaluating the possible impact of extrasystoles and atrial fibrillation on the visibility of proximal segments, generalized linear models did not show a significant difference in visibility by the presence of atrial fibrillation (RD 0.02; 95% CI −0.13 to 0.17) or extrasystoles (RD −0.03; 95% CI −0.22 to 0.16). Conversely, visibility depended on heart rate, which was significantly lower in the visible segments (difference −11 bpm, 95% CI −18 to −4).

An empirical cut-off of 75 beats per minute provided a sensitivity of 86% for lack of visualization of proximal coronary arteries and a specificity of 56%, with an AUROC of 0.71.

For heart rates of 75 beats per minute or less, the percentage of visible proximal coronary arteries was 97%. In contrast, above the cut-off it was 82%, with a statistically significant difference between the two groups (RD 15%, 95% CI 2–28%). Only one patient had a significant lesion and underwent coronary artery bypass grafting (CABG) during surgery. One patient required invasive angiography after CTA.

### 3.3. Agreement

Cardiac lesions consistent with IE and vegetations, as determined by radiological modalities or surgical inspection, are summarized in Table 2.

TOE images were not available for 4 patients because transthoracic examinations were performed only. Nevertheless, we decided not to include transthoracic findings due to the different sensitivity of the methods. Vegetations were described in 21 patients with TOE, in 12 patients with CCT and in 13 cases during surgical inspection. Mean vegetation size was 8.28 mm in TOE and was higher than CCT (3.93 mm). For surgical inspection, the vegetation size was not available since it is not usually reported. About paravalvular complications, abscesses were frequently detected only on surgical inspection, as only 2 of the 11 cases described in surgical reports were detected by CCT and 5 by TOE. The presence of a pseudoaneurysm, described in 9 cases by CCT and TOE, was confirmed during surgical inspection in 3 cases.

**Figure 3:**
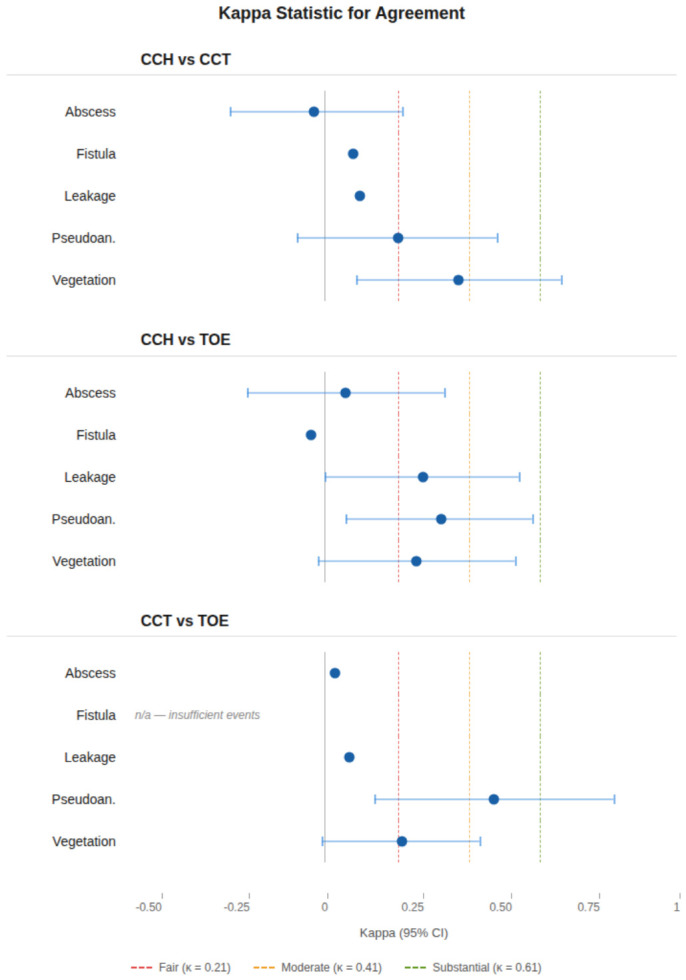
Forrest plot reporting the kappa statistic for each of the examined variables (dot) and 95% CI (whisker). Dashed vertical lines stand for the lower limit for fair agreement (red); moderate agreement (orange) and substantial agreement (green); CCH: cardiac surgery; CCT: cardiac computed tomography; TOE: transoesophageal echocardiogram.

The agreement between CCT, TOE, and surgery (CCH) is illustrated in Figure 3.

The agreement between CCT and TOE was only slight for vegetations (kappa 0.14) and abscesses (kappa 0.0), while it was fair for pseudoaneurysms (kappa 0.46) and paravalvular leakage (kappa 0.31).

The agreement between CCT and surgery was poor for abscesses (kappa 0.0), while it was fair for vegetations (kappa 0.36) and pseudoaneurysms (kappa 0.36) and moderate for paravalvular leakage (kappa 0.46).

The agreement between surgery and TOE was fair for pseudoaneurysms (kappa 0.46) and vegetations (kappa 0.24), but poor for abscesses (kappa 0.06) and paravalvular leakage (kappa −0.15).

### 3.4. Safety

One patient had a mild allergic reaction and developed urticaria after contrast enhancement injection, treated with chlorpheniramine maleate and methylprednisolone. One patient had a history of previous iodine allergic reaction and did not develop any allergic reaction after preparation according to local standard procedures.

Median creatinine levels before cardiac CT were 0.96 mg/dL (IQR 0.8–1.27 mg/mL), with an eGFR of 87.21 mL/min (IQR 49–97.40 mL/min). Three of the included patients (9.09%) developed stage 1 AKI within 72 h of cardiac CT according to KDIGO classification.

## 4. Discussion

In our cohort of patients with AVIE undergoing CCT, visualization of the coronary arteries was adequate for the proximal segments but poor for the medial and distal segments.

While proximal coronary arteries and LAD 2 were visible in almost every patient, visibility of the distal and median segments was generally poor.

Visualization of coronary arteries using ECG-gated CTA depends on scan resolution and heart rate [9]. Particularly, a heart rate below 62 bpm is associated with improved sensitivity. In our cohort, the median heart rate was 73 bpm, which explains the low performance for distal coronary segments.

This difference in heart rate may be due to a different indication: CCT was primarily performed to evaluate the presence of IE-specific lesions at the valvular and aortic root levels, which can often be adequately assessed even at higher heart rates.

With this in mind, we found that proximal segments were likely unevaluable at heart rates above 78 bpm, indicating a high NPV for proximal segment visualization with this empirical heart rate cut-off.

Since ESC guidelines recommend CTA for CAD evaluation in patients with IE who have a surgical indication and cardiovascular risk factors, this finding has relevant clinical implications [3].

In fact, CTA has shown an excellent negative predictive value for ruling out stable CAD. Still, its diagnostic performance is reduced by unevaluable results and is influenced by pre-test probability [9].

Overall, patients with AVIE are at lower risk of CAD than those with other valvular diseases [7,14], as shown in this cohort, in which only 1 patient underwent CABG.

Therefore, risk stratification for CAD is crucial. In lower-risk patients, the probability of missing the disease despite suboptimal CTA results is low. In this scenario, the clinical impact of limited distal evaluation may be negligible.

On the other hand, an inadequate CTA may miss significant lesions and may require further angiography [7] in patients at higher risk.

In a large cohort of patients undergoing CAD evaluation for AVIE, Knol et al. found that 13% of patients required downstream invasive angiography after CTA, concluding that CTA could serve as a gatekeeper for invasive angiography [7].

Performing angiography after CTA may increase the risk for contrast-induced acute kidney injury [7,12]. While in our cohort mild kidney injury occurred in a limited proportion of patients, patients with IE can experience kidney failure for several reasons, and AKI has been associated with worse outcomes [1]. Therefore, the risk of further nephrotoxicity should be considered in the decision-making algorithm.

In our cohort, only one patient required invasive angiography after CTA. While this finding could suggest a limited clinical impact of suboptimal distal evaluation, it should be interpreted cautiously. This was a single-center cohort study with low CAD prevalence, and the results cannot be generalized to settings with higher prevalence [7].

Invasive angiography is usually contraindicated in AVIE due to the risk of coronary artery embolization. Still, several small cohort studies have shown that this procedure is safe in patients without large (>10 mm) and mobile vegetations [3,6,7].

Regarding concordance among CCT, TOE, and cardiac surgery, agreement on lesions consistent with IE was overall low.

Studies evaluating the diagnostic performance of CCT and TOE for IE have consistently shown higher TOE sensitivity for vegetations and leaflet perforation [5,15,16]. This is reflected in this cohort, where vegetations were detected by echocardiography in 87% of patients, but only in nearly half of the cases by CCT or during surgical inspection. Similarly, only one of nine leaflet perforations described by TOE was confirmed by CCT.

Interestingly, we found that the median vegetation size was lower in CCT than in TOE. Despite being apparently at odds with the higher resolution of TOE for small vegetation [4,5], this finding can be explained by the median six-day interval between the two exams. Since CCT is performed after TOE, vegetations could have been reduced due to antimicrobial treatment or peripheral embolization. This could also explain the lower proportion of vegetations detected during surgery, as patients with non-urgent surgical indications may have undergone operation after several days of antimicrobial treatment, allowing resolution of vegetations, particularly smaller ones.

While the number of paravalvular complications detected was similar between TOE and CCT, the interpretation of lesion type differed between the two modalities and from intraoperative findings.

The time interval between TOE and CCT can partially explain discrepancies among diagnostic techniques, given the dynamic evolution of paravalvular complications.

Surgical inspection confirmed the presence of pseudoaneurysms detected by TOE and CCT in only 3 of 9 patients. This difference may be due to the misclassification of other paravalvular complications. On the other hand, abscesses were detected only during surgical inspection, with CCT used in only 2 of the 11 cases described in surgical reports and TOE in 5 of 11.

This finding is consistent with previous studies in a cohort of patients with AVIE and perivalvular abscesses, in which 25% of aortic root abscesses were detected only during surgery [17].

In our cohort, CCT detected paravalvular leakage in four cases, compared with two detected by TOE. These findings apparently contrast with what has been previously described.

A large systematic review of the performance of CCT and TOE in IE by Jain et al. showed that TOE had 100% sensitivity for paravalvular leakage, compared with 85% for CCT. Nevertheless, the study was underpowered to detect a statistically significant difference, as stated by the authors [5]. Therefore, the performance of the two exams in detecting this type of lesion may be similar. Furthermore, our findings are purely descriptive due to the limited number of events.

Agreement between CCT and TOE was low to moderate across all lesion types. A possible interpretation of this finding is that CCT plays a complementary role in the diagnosis of IE, particularly when prosthetic valves are involved or when paravalvular complications are suspected [16,18].

Moreover, CCT has shown prognostic value in patients with IE undergoing surgery, as detecting pseudoaneurysms and abscesses is associated with increased mortality and provides useful information for surgical planning [4].

One of the main strengths of the present study is the detailed evaluation of coronary segment visualization, which highlights the possible limitations of CTA for CAD screening in patients with AVIE. Moreover, this study focused on the concordance between TOE and CCT, which has previously been poorly described, thereby supporting a potential complementary role of CCT in the evaluation of IE-specific lesions.

Nevertheless, several limitations should be acknowledged.

The small sample size and limited number of events reduce the statistical power of the concordance evaluation and the generalizability of the results; similarly, the single-center design limits the external validity of the results. For example, findings related to the need for downstream invasive angiography should be tested in cohorts with higher CAD prevalence.

Therefore, these findings should be considered hypothesis-generating rather than definitive.

This study was conducted as a retrospective evaluation of a prospective cohort of all consecutive patients with IE. Nonetheless, selection bias probably occurred. Due to a lack of standardized indication, CCTs were performed at clinicians’ discretion. Similarly, since CCT provides useful information for surgical plans, the proportion of patients undergoing surgery is overrepresented.

Another limitation is due to the lack of formal interobserver agreement analysis. The absence of reproducibility assessment may have influenced diagnostic agreement estimates.

Furthermore, the presence of missing data, particularly for TOE findings, due to the retrospective nature of the study, and the limited number of events, could underestimate the concordance between diagnostic techniques.

Reduction in vegetation size due to antimicrobial therapy and embolization, or the evolution of paravalvular complications, can partially explain the low concordance among diagnostic techniques and represent an important confounder.

Finally, our evaluation spans a long period, and the results may be influenced by the increased use of CCT for patients with AVIE, thereby enabling more accurate patient selection and preparation.

## 5. Conclusions

While the results of this study suggest a complementary role for CCT in the diagnosis of IE, these findings should be interpreted with caution. Low concordance may be explained by factors other than complementarity, particularly the limited sample size, limited number of events, temporal changes, and interobserver variability.

Visibility of the distal coronary arteries was suboptimal. While this performance may be acceptable in most low-risk patients with IE, the risk of missing significant lesions in high-risk patients should be acknowledged.

Therefore, the CTA indication should consider several factors, such as the presence of CAD risk factors, the feasibility of slowing the heart rate to ensure adequate coronary artery evaluation, and the embolic risk associated with invasive angiography.

The risks and benefits of using β-blockers and nitroglycerin, which are suggested to improve coronary artery evaluation, should be carefully weighed for each individual patient [18].

With this in mind, a multidisciplinary approach within the endocarditis team seems crucial to improve patient selection and individual outcomes in AVIE.

Prospects for improving the diagnostic role of CCT in IE could include new technologies, particularly photon-counting cardiac computed tomography (PCCT), which has shown improved coronary artery resolution and reduced metal artifacts from prosthetic valves [19,20].

## Figures and Tables

**Table 1:** Patients’ demographic and clinical characteristics. Categorical variables are expressed as counts and percentages, while continuous variables are expressed as medians and IQRs.

Variable		Variable	
Sex, female	6 (15.38)	**IE complications**	
Age	65 (54–73)	Heart failure	9 (23.68)
**IE Risk factors**		Cerebral emboli	10 (25.64)
CV procedures	19 (48.72)	Arrhythmias	6 (15.38)
IVDU	3 (7.69)	**Echocardiography**	
Anatomical abnormalities	5 (12.82)	Heart rate during TOE	72 (65–85)
GI tract foci	6 (15.38)	EF left ventricle, %	60.5 (55–64)
**Comorbidities**		Left ventricle volume, mm	157.5 (122.5–190)
Ischemic heart disease	3 (7.69)	Time between TOE and CCT, days	6 (3–75)
Previous stroke	3 (7.69)	**Cardiac CT**	
Vascular disease	8 (21.62)	Creatinine before CCT, mg/dL	0.96 (0.8–1.27)
COPD	2 (5.41)	eGFR before CCT, mL/min	87.21 (49–97.40)
CKD	6 (16.22)	CCT-related AKI	3 (9.09)
AF	3 (8.11)	Heart rate during CT	74 (64–86)
DM	8 (21.62)	AF/extrasystoles during CT	2 (5.13)
Smoke	13 (36.11)	Time between CCT and surgery, days	6 (2–14)
Hypercholesterolemia	6 (16.67)		
Previous cardiac surgery	21 (53.85)		
Previous stent	3 (7.69)		
Previous bypass	3 (7.69)		
Prosthetic valve	20 (52.63)		

Abbreviations: IE = infective endocarditis, CV = cardiovascular, IVDU = intravenous drug users, GI = gastrointestinal, COPD = chronic obstructive pulmonary disease, CKD = chronic kidney disease, AF = atrial fibrillation, DM = diabetes mellitus, EF = ejection fraction, TOE = transoesophageal echocardiography, CCT = cardiac CT, eGFR = estimated glomerular filtration rate, AKI = acute kidney injury.

**Table 2:** Lesions consistent with IE detected by cardiac CT, transoesophageal echocardiography and surgical inspection.

Lesion	CCT (*n* = 28)	TOE (*n* = 24)	Cardiac Surgery (*n* = 28)
Vegetations	12 (42.86)	21 (87.50)	13 (46.4)
Vegetation size, mm	3.93 ± 5.23	8.28 ± 3.92	
Leaflet perforation	1 (3.57)	9 (37.50)	8 (28.6)
Paravalvular complications	11 (39.29)	13 (54.17)	15 (53.6)
Abscesses	2 (7.14)	5 (20.83)	11 (39.3)
Pseudoaneurysm	9 (32.14)	9 (37.50)	3 (10.7)
Paravalvular leakage	4 (14.29)	2 (8.33)	5 (17.84)
Fistula	0 (0.00)	1 (4.17)	2 (7.1)

Categorical variables are expressed as counts and percentages, while continuous variables are expressed as means and standard deviations. Vegetation size was not reported for surgical inspection. CCT = cardiac CT, TOE = Transoesophageal echocardiography.

## Data Availability

The data presented in this study are available on request from the corresponding author due to privacy and ethical restrictions.

## Supplementary Materials

The following supporting information can be downloaded at: https://www.mdpi.com/article/10.3390/diagnostics16142160/s1, Table S1. Causative organisms and antimicrobial treatments for the included endocarditis episodes.
