# Supplementary material for: Implementation of Cardiac Computed Tomography for Aortic Valve Infective Endocarditis: Coronary Arteries Evaluation and Concordance with Transoesophageal Findings
Source: Diagnostics (Basel). 2026 Jul 10;16(14):2160. doi: 10.3390/diagnostics16142160 (PMC13408644; doi:10.3390/diagnostics16142160)
Supplement: Supplementary file 1 [file diagnostics-16-02160-s001.zip › diagnostics-4311527-supplementary.pdf]

**Table S1 Causative organisms and antimicrobial treatments for the included endocarditis episodes**			
Isolate		Antimicrobial treatment	
MSSA	6 (15.38)	Daptomycin/Ampicillin	2 (5.13)
MRSA	1 (2.56)	Daptomycin/Cefazolin	5 (12.82)
E. faecalis	12 (30.77)	Daptomycin/Ceftaroline	3 (7.69)
Streptococcus spp	9 (23.08)	Daptomycin/Ceftriaxone	1 (2.56)
CoNS	8 (20.51)	Daptomycin/Oxacillin	4 (10.26)
Gemella morbillorum	1 (2.56)	Daptomycin/Fosfomycin	1 (2.56)
Polimicrobial	1 (2.56)	Vancomycin/Amoxicillin	1 (2.56)
Colture Negative	1 (2.56)	Vancomycin/Ceftriaxone	3 (7.69)
		Cefazolin	1 (2.56)
		Ceftriaxone	6 (15.38)
		Ampicillin/Ceftriaxone	9 (23.08)
		Ampicillin/Gentamycin	2 (5.13)
		Ceftriaxone/Gentamycin	1 (2.56)
Results are expressed as counts and percentages. MSSA=methicillin susceptible Staphylococcus aureus, MRSA= methicillin resistant Staphylococcus aureus, CoNS= coagulase negative staphylococci			
